# Tonsil-derived mesenchymal stem cells exert immunosuppressive effects on T cells

**DOI:** 10.3325/cmj.2019.60.12

**Published:** 2019-02

**Authors:** Antun Bačić, Drago Prgomet, Saša Janjanin

**Affiliations:** 1 Clinical Department of Ear, Nose and Throat (ENT), Head and Neck Surgery, Merkur University Hospital, Zagreb, Croatia; 2 University Department of Ear, Nose and Throat (ENT), Head and Neck Surgery, Zagreb University Hospital Center, Zagreb, Croatia; 3 Dubai Healthcare City, Dubai, United Arab Emirates

## Abstract

**Aim:**

To assess the immunomodulatory effect of tonsil-derived mesenchymal stem cells (MSCs) on T-lymphocyte proliferation and cytokine production.

**Methods:**

Tonsils were obtained from children aged 3 to 12 years (n = 15) who underwent tonsillectomy for obstructive sleep apnea from April 2012-October 2014 at the Merkur University Hospital, Zagreb. Tonsil-derived MSCs were co-cultured with peripheral blood mononuclear cells (PBMCs) and phytohemagglutinin as a mitogen. PBMCs were induced to differentiate into T helper 1 or T helper 2 cells in the presence or absence of tonsil-derived MSCs, after which the production of interferon-gamma in T helper 1 and interleukin-4 in T helper 2 cells was assessed.

**Results:**

Tonsil-derived MSC suppressed phytohemagglutinin-induced proliferation of PBMCs. Compared with controls, tonsil-derived MSC co-culture significantly decreased interferon-gamma production (*P* < 0.001) and increased interleukin-4 production (*P* < 0.001).

**Conclusion:**

Tonsil-derived MSCs exert immunomodulatory effects on T lymphocyte proliferation and T helper 1- and T helper 2-specific cytokine production.

Multipotent mesenchymal stem cells (MSCs) differentiate into various tissues of mesodermal origin, including bone, cartilage, and adipose and muscle tissues (1). Although MSCs do not induce immune response in an immunocompetent alloreactive host, in immunocompromised individuals they suppress T-, B-, natural killer, and dendritic cell proliferation and function (2-4). Several studies demonstrated the immunosuppressive properties of MSCs, highlighting the inhibition of T cell proliferation as their most important effect (2,5). MSCs also suppress other T cell functions, such as activation, cytokine secretion, and cytotoxicity. They inhibit the expression of T cell surface activation markers, antigen-specific T cell maturation, formation of cytotoxic T-lymphocytes, and production of proinflammatory interferon-gamma (IFN-γ) in T helper 1 (Th1) cells and immunosuppressive interleukin-4 (IL-4) in T helper 2 (Th2) cells (6,7).

MSCs for research and therapeutic use are mostly isolated from bone marrow; however, they can also be successfully isolated from many other adult tissues, including palatine tonsils (8-10). Palatine tonsils are an important alternative source of mesenchymal progenitor cells because of simple surgical access, lack of ethical dilemmas on tonsillectomy, and the fact that tonsils are generally removed in children and young adults, which provides young cells with high proliferation potential. In addition, since tonsillectomy is a common surgical procedure in children and adults, palatine tonsils are one of the most abundant surgical residual tissues (11). Previous studies have demonstrated human palatine tonsils to be a good source of MSCs (8). Furthermore, protocols for MSC isolation and their cell culture conditions have been established, the cell surface markers have been characterized, and MSC differentiation into mesodermal tissues has been demonstrated (8).

Considering that tonsil-derived MSCs have comparable phenotypic properties and differentiation characteristics to MSCs isolated from other tissues, we hypothesized that they may also exhibit immunomodulatory properties. Therefore, we assessed the immunomodulatory effect of tonsil-derived MSCs on T-lymphocyte proliferation and specific T-lymphocyte cytokine production *in vitro*.

## Material and methods

### T-MSC isolation

Tonsils were obtained from children aged 3 to 12 years (n = 15) who underwent tonsillectomy for obstructive sleep apnea in the period April 2012-October 2014 at the Merkur University Hospital but had no other metabolic, cardiac, or respiratory diseases. The study was approved by the institutional review board of the Merkur University Hospital (KBM 03/11/1259, date of approval: March 17, 2011), and parents signed the informed consent for study participation and data publication.

Tonsils were de-epithelialized and digested in the Roswell Park Memorial Institute (RPMI)-1640 medium (Invitrogen, Carlsbad, CA, USA) containing 210 U/mL collagenase type I (Invitrogen) and 90 kU/mL DNase I (Sigma-Aldrich, St. Louis, MO, USA) for 30 minutes at 37°C. The cells were washed twice in 20% normal human serum (NHS)-RPMI and once with 10% NHS-RPMI after filtration through a wire mesh. The supernatant was discarded after centrifugation (centrifuged for 10 minutes at 4000 RPM), and the cell pellet was resuspended in 50 mL phosphate buffered saline (PBS); after dilution, 10 mL suspension was distributed in five polypropylene tubes. Mononuclear cells were isolated by Ficoll-Paque (Amersham, GE Healthcare, Little Chalfont, Buckinghamshire, UK) density gradient centrifugation of processed cell pellet, which was plated for 24 to 48 hours in T-150 tissue culture flasks (Corning Incorporated, Corning, NY, USA). Non-adherent cells were subsequently removed by flushing with serum-free MSC expansion medium (R&D Systems, Minneapolis, MN, USA).

### T-MSC expansion

Serial passages of the cell culture were performed when tonsil-derived MSCs attained 80% confluence (as determined by phase contrast microscopy). Cells were washed twice with PBS, detached using 0.25% trypsin-EDTA solution (Gibco-BRL, Carlsbad, CA, USA), washed twice with expansion medium using centrifugation (1200 RPM for 5 minutes), and replated at 1:3 dilution under the same culture conditions. Cells obtained at passages 2 to 5 were used.

### Cell surface epitope profiling – flow cytometry analysis

Tonsil-derived MSCs (>1 × 10^5^ cells) were harvested and mixed with PBS containing 1% fetal bovine serum (FBS) (P+F mixture) with dilutions (1:100) of subsequent conjugated mouse IgG1κ anti-human monoclonal antibodies: CD14-phycoerythrin (PE), CD31-PE, CD45- fluorescein isothiocyanate (FITC), CD73-PE, CD90-FITC, CD105-PE (BD Biosciences, San Jose, CA, USA), HLA-A, B, C-PE (MHC I), HLA-DR, DP, DQ-FITC (MHC II) (R&D Systems) over 1 hour (at 4°C). Cell suspensions were washed twice with P+F and resuspended in P+F for analysis on a flow cytometer (FACSCalibur, BD Biosciences) using the CellQuest Pro^TM^ software (BD Biosciences). Positive staining was defined as fluorescence emission higher than the levels obtained in more than 99% of the cells in a population stained with isotype controls.

### ***In vitro*** differentiation

Tonsil-derived MSCs were induced for adipogenic, osteogenic, and chondrogenic differentiation. For adipogenic differentiation, cells were incubated for 3 weeks in Dulbecco's modified Eagle's medium (DMEM) supplemented with 10% FBS, 1 μM dexamethasone, 1 μg/mL insulin, and 0.5 mM 3-isobutyl-1-methylxanthine (all Sigma-Aldrich). For osteogenic differentiation, cells were incubated for 3 weeks in DMEM supplemented with 10% FBS, 10 nM dexamethasone, 50 μg/mL ascorbic acid-2-phosphate, 10 mM β-glycerophosphate, and 10 nM 1,25 dihydroxyvitamin D_3_ (Biomol International L.P., Plymouth Meeting, PA, USA). For chondrogenic differentiation, pelleted cultures were incubated for 3 weeks in high-glucose DMEM supplemented with 100 nM dexamethasone, 40 μg/mL L-proline, 100 μg/mL sodium pyruvate, 50 μg/mL ascorbic acid-2-phosphate, 10 ng/mL recombinant human transforming growth factor-β3 (R & D Systems), and 50 mg/mL insulin-transferrin-selenium-premix stock (BD Biosciences).

### Total RNA isolation and quantitative reverse transcription polymerase chain reaction (qRT-PCR)

Total RNA samples were obtained from tonsil-derived MSCs after 3 weeks of culture using Trizol reagent (Invitrogen Corporation) and reverse-transcribed using random hexamers. Ten nanograms of complementary DNA (cDNA) and SYBR Green mix (Bio-Rad Laboratories, Irvine, CA, USA) was used for qRT-PCR with gene-specific primers (forward/reverse) designed using GenBank cDNA sequences (Table 1). Specific transcript levels were normalized to that of glyceraldehyde-3-phosphate dehydrogenase (GAPDH) and presented as fold increase over GAPDH levels using the 2^(ΔCt)^ method, where ΔCt = Ct of target gene - Ct of GAPDH.

**Table 1:** Primers for reverse transcription-polymerase chain reaction of differentiation-specific genes

Gene*	Primer sequences (5′–3′)	Position (bp)	Predicted size (bp)
Housekeeping gene			
*GAPDH*	Sense: GGGCTGCTTTTAACTCTGGT Antisense: GCAGGTTTTTCTAGACGG	134–835	702
Adipose tissue-specific genes			
*LPL*	Sense: GAGATTTCTCTGTATGGCACC Antisense: CTGCAAATGAGACACTTTCTC	1261–1536	276
*PPARγ*	Sense: GCTGTTATGGGTGAAACTCTG Antisense: ATAAGGTGGAGATGCAGGCTC	153–503	351
Bone-specific genes			
*ALP*	Sense: TGGAGCTTCAGAAGCTCAACACCA Antisense: ATCTCGTTGTCTGAGTACCAGTCC	122–575	454
*OC*	Sense: ATGAGAGCCCTCACACTCCTC Antisense: GCCGTAGAAGCGCCGATAGGC	19–312	294
Cartilage-specific genes			
*AGN*	Sense: TGAGGAGGGCTGGAACAAGTACC Antisense: GGAGGTGGTAATTGCAGGGAACA	6591–6910	350
*COL2A1*	Sense: CAGGTCAAGATGGTC Antisense: TTCAGCACCTGTCTCACCA	1341–1717	377

*GAPDH – glyceraldehyde-3-phosphate dehydrogenase; LPL – *lipoprotein lipase*; *PPARγ* – *peroxisome proliferator-activated receptor-γ*; *ALP* – *alkaline phosphatase*; *OC* – *osteocalcin*; *AGN* – *aggrecan*; *COL2A1* – *collagen type II α1*.

### Primary mixed lymphocyte reaction

Peripheral blood was obtained from healthy human donors (n = 15). Peripheral blood mononuclear cells (PBMCs) were segregated by using Ficoll-Hypaque density gradient centrifugation and subsequently mixed with RPMI-1640 medium containing 10% FBS, 100 μg/mL streptomycin, 100 U/mL penicillin, 2 mM glutamine, 0.1 mM non-essential amino acids, 1 mM sodium pyruvate, 20 mM HEPES, and 50 μM 2-mercaptoethanol. 1 × 10^5^ PBMCs/100 μL (in triplicates) were seeded in 96-well round-bottom plates (BD Biosciences). T cell activator phytohemagglutinin was used as a positive control mitogen (5 μg/mL). 5 × 10^4^ of tonsil-derived MSCs were added to obtain a final volume of suspension of 300 μL per well. Plates were incubated for 72 hours, after which 1 μCi/[^3^H]-thymidine (GE Healthcare) was added to each well. After overnight incubation, incorporation of radioactivity was measured by liquid scintillation counting. All tests were done in triplicates and repeated at least twice.

### MSCs-induced T lymphocyte co-culture

Tonsil-derived MSCs and PBMCs were co-cultured in 12-well plates (tonsil-derived MSCs to PBMCs ratio, 1:10) under Th1- or Th2-inducing conditions. Control plates contained tonsil-derived MSCs or PBMCs only. Th1-inducing medium contained anti-CD3 [5 μg/mL], anti-CD28 [1μg/mL], recombinant human IL-2 [rhIL-2, 4 ng/mL], rhIL-12 [1 μg/mL], and anti-IL-4 [1μg/mL]. Th2-inducing medium contained anti-CD3 [5 μg/mL], anti-CD28 [1 μg/mL], rhIL-2 [4 ng/mL], rhIL-4 [1 μg/mL], and anti-IFN-γ [1 μg/mL] (Sigma-Aldrich). After two days of incubation, the non-adherent cells were collected from co-cultures and mixed with phytohemagglutinin (5 μg/mL) for further 24 hours. The levels of IFN-γ in Th1 and IL-4 in Th2 co-cultures were analyzed using commercial enzyme-linked immunosobent assay kits (R&D Systems) as per manufacturer’s instructions and compared with controls.

### Statistical analysis

All data are presented as mean ± standard deviation unless otherwise indicated. The sample size was based on literature search and is similar to that in other studies (3,7). The study power was calculated from sample size, effect size (Cohen's d), and significance level. The lowest power was obtained for the assessment of IFN-γ secretion. For IFN-γ levels, the Cohen's d was 2.9 (signifying the large effect), so the significance level was set at 0.01 to correct for multiple comparisons (since there were three groups). The calculated power based on these data was at least 85%, while the power for other comparisons was even higher.

Normality of data distribution was tested by the Kolmogorov-Smirnov test. The differences between the groups in data from flow cytometry analysis of surface epitope profiles and real-time PCR analysis of differentiation-specific genes were assessed with the *t* test. The differences in cytokine secretion were assessed with one-way ANOVA with Bonferroni *post hoc* correction. The significance level was set at *P* < 0.05. Statistical analysis was performed with the SPSS software, v. 23 (IBM Corp., Armonk, NY, USA).

## Results

### Cell isolation and proliferation

**Figure 1:**
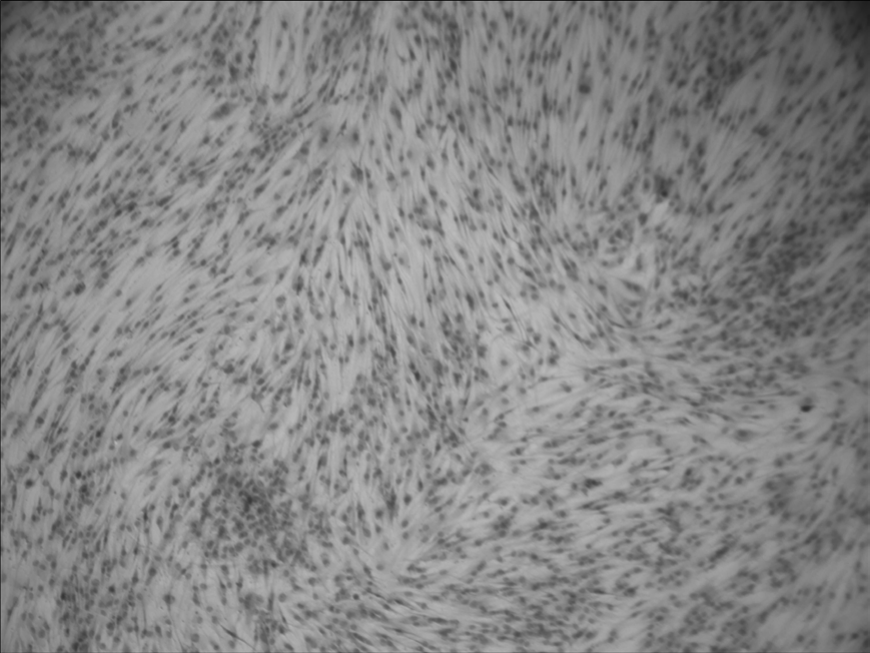
Microscopic view of unstained fibroblast-like tonsil-derived mesenchymal stem cells with flat polygonal morphology at passage 1. Magnification: 40 × .

The total cell yield from each tonsil (n = 15) was in the range of 1-5 × 10^9^. The majority of these cells were non-adherent; most likely of hematopoietic origin. Approximately 0.1%-1% of the isolated cells remained adherent to flasks after repetitive irrigations with PBS and expansion-medium changes. Tonsil-derived MSCs were homogenous fibroblast-like with extended cytoplasmic processes (Figure 1).

### Flow cytometry analysis of surface epitope profiles

**Figure 2:**
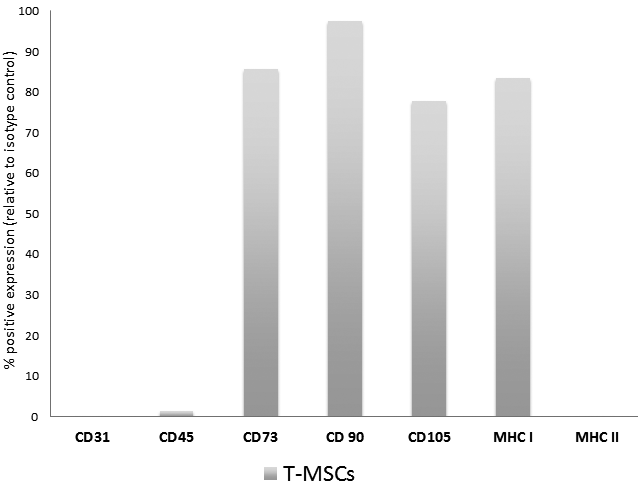
Flow cytometry analysis of tonsil-derived mesenchymal stem cells (T-MSC). Cluster of differentiation (CD)14, CD34, CD45, CD73, CD90, CD105, major histocompatibility complex (MHC) I, and MHC II were analyzed by fluorescent conjugated antibodies. The expression of each epitope is presented as the percentage of positive expression relative to the isotype control.

Flow cytometric analysis showed non-hematopoietic and non-endothelial origin of tonsil-derived MSCs as they did not express CD45 and CD31. However, they were positive for characteristic MSC surface epitope markers, such as CD105, CD73, and CD90 (Figure 2). They also expressed MHC class I but not MHC class II molecules.

### Multilineage differentiation potential

**Figure 3:**
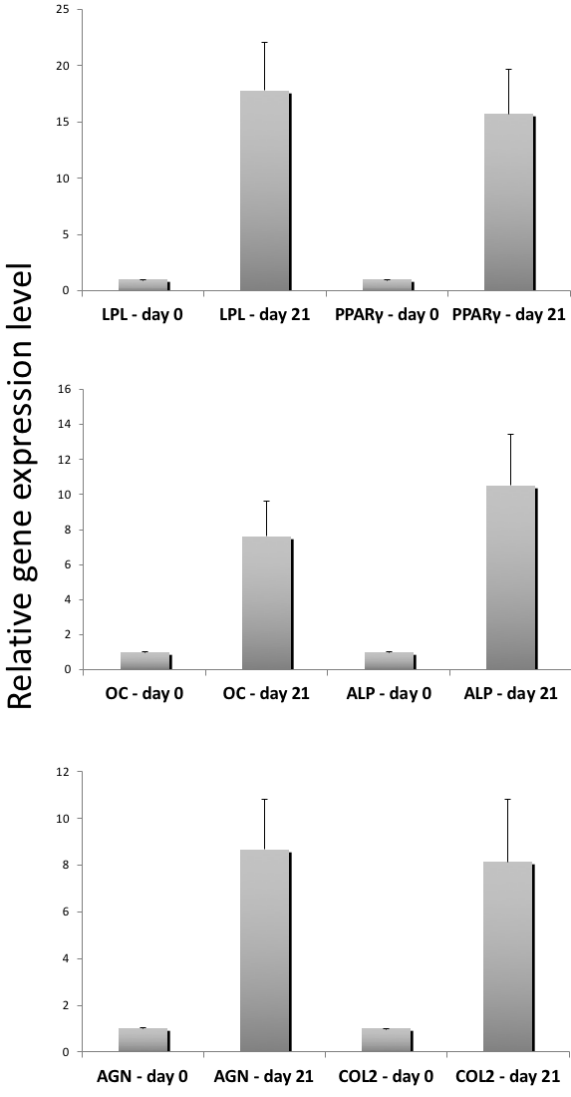
Gene expression analysis of differentiated tonsil-derived mesenchymal stem cells. *Lipoprotein lipase* (*LPL*) and *proliferation-activated receptor-gamma* (*PPARγ*) are genes involved in adipogenesis, *osteocalcin* (*OC*) and *alkaline phosphatase* (*ALP*) are genes involved in osteogenesis, and *aggrecan* (*AGN*) and *collagen type II α1* (*COL2*) are genes involved in chondrogenesis. Gene expression analysis was performed at the beginning of culture (day 0) and at day 21. Expression levels were normalized to that of glyceraldehyde 3-phosphate dehydrogenase (GAPDH), and the results are reported as marker gene vs GAPDH ratios using the formula 2ΔCT ( × 100). Values are mean ± standard deviation (n = 2). Error bars represent standard deviation.

Tonsil-derived MSCs in passages 2-5 were treated with adipogenic, osteogenic, and chondrogenic supplements. After 3 weeks of culture, qRT-PCR analysis of the differentiated cells revealed a significant increase in *lipoprotein lipase* and *peroxisome proliferator-activated receptor-γ* expression in adipogenic cultures; *osteocalcin* and *alkaline phosphatase* expression in osteogenic cultures; and *aggrecan* and *collagen type II α1* expression in chondrogenic cultures (Figure 3).

### Inhibition of T cell proliferation

**Figure 4:**
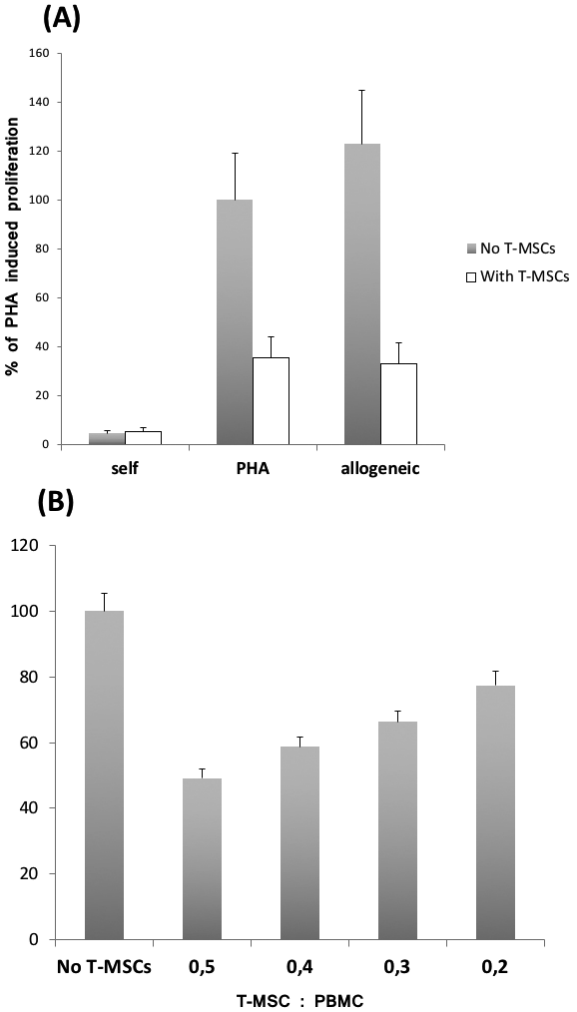
Tonsil-derived mesenchymal stem cells (T-MSCs) inhibited phytohemagglutinin (PHA)-stimulated T cell proliferation in a dose-dependent manner. Peripheral blood mononuclear cells (10^5^ cells) with 5 μg/mL PHA were incubated for 72 hours in the absence or presence of T-MSCs (5 × 10^4^ or varying ratios). (**A**) Cell proliferation based on [3H]-thymidine uptake. T-MSCs inhibited the T cell proliferation. The value of 100% was set at the proliferative response (counts per minute per culture) of PHA-stimulated T cell proliferation. All values are mean ± standard deviation of triplicates. (**B**) T-MSCs demonstrated a dose-dependent suppression of PHA-stimulated T cell proliferation. Results (n = 3) are expressed as percentage relative to T cell proliferation without addition of T-MSCs (the value of 100%). Error bars represent standard deviation. Gray bars – without T-MSCs; white bars – with T-MSCs.

To assess the immunomodulatory effect of tonsil-derived MSCs on T cell responses, we first used *in vitro* assays of proliferative T cell activity with phytohemagglutinin as a mitogen. Tonsil-derived MSC addition to cultured and phytohemagglutinin-stimulated PBMCs robustly inhibited PBMC proliferation (Figure 4A). We observed a dose-dependent effect, except at 1:5 tonsil-derived MSC-to-PBMC ratio, when the effect of tonsil-derived MSCs was largely absent (Figure 4B). Paired *t* test showed that the immunosuppressive effect of tonsil-derived MSCs on phytohemagglutinin-stimulated proliferation of T cells was significant (*P* < 0.001). There was no significant self-induced effect (*P* = 0.150)

### MSC-T cell interaction

**Figure 5:**
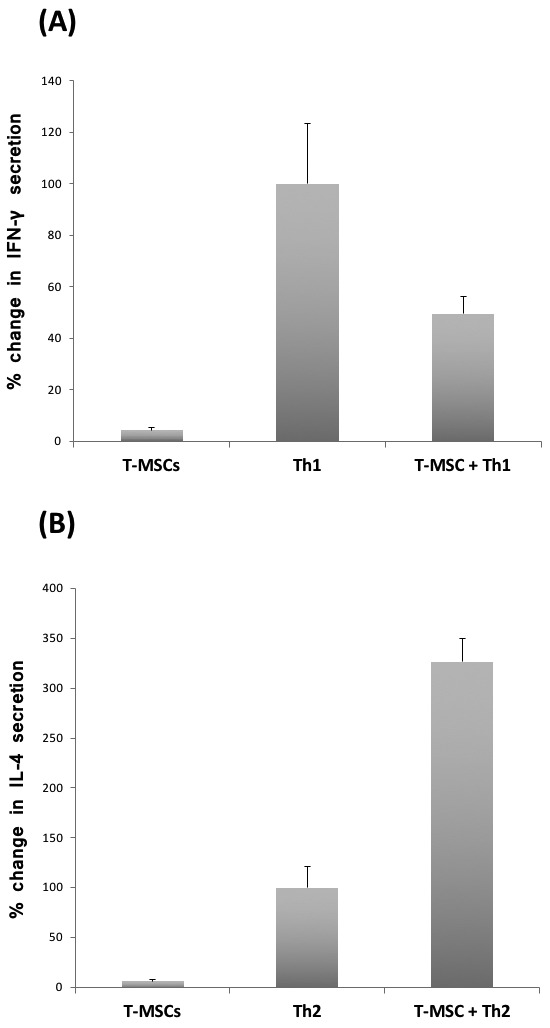
Tonsil-derived mesenchymal stem cells (T-MSCs) were co-cultured with peripheral blood mononuclear cells under T helper 1 (Th1) or T helper 2 (Th2)-inducing conditions. (**A**) In the presence of T-MSCs, Th1 cells showed >50% decrease in interferon-gamma (IFN-γ) production and (**B**) Th2 showed >300% increase in interleukin-4 (IL-4) production compared with controls without T-MSCs. Values indicate the change in cytokine secretion (mean ± standard deviation) in the absence or presence of T-MSCs in three independent experiments. Error bars represent standard deviation.

We specifically focused on the production of Th1- and Th2-specific cytokines in T cells generated under polarizing *in vitro* conditions. PBMCs undergoing Th1 differentiation without addition of tonsil-derived MSCs produced moderate levels of IFN-γ. After the addition of tonsil-derived MSCs, these levels significantly decreased (*P* < 0.001) (Figure 5A). In Th2 cells, addition of tonsil-derived MSCs significantly increased IL-4 production (*P* < 0.001) when compared with baseline IL-4 levels.

## Discussion

Our study identified a potent immunomodulatory effect of human tonsil-derived MSCs on proliferative and cytokine production. In the presence of tonsil-derived MSCs, T cells exhibited significantly decreased proliferation, decreased IFN-γ production, and increased IL-4 production, which differs from the typical inflammatory profile.

Our findings extend the results of earlier studies that showed that other MSCs (bone marrow, Wharton's jelly, and fat tissue) exerted immunomodulatory effects on CD4^+^ and CD8^+^ T cells (12) and that tonsil-derived MSCs in mouse models modulated human immune responses (13).

Compared with controls, we observed >50% decrease in IFN-γ secretion in Th1 cells and >300% increase in IL-4 production in Th2 cells when they were co-cultured with tonsil-derived MSCs. This is similar to other studies that showed that MSCs can alter immune cell response by inhibiting proinflammatory TNF-α and IFN-γ and increasing anti-inflammatory IL-4 and IL-10 secretion (6).

Both autologous and allogeneic MSCs suppress CD4^+^ Th cells and CD8^+^ cytotoxic T-lymphocytes (14,15). This suppression of T cells may be direct, or indirect through modulatory action on antigen-presenting cells (16). Another mechanism is the arrest in the G_0_/G_1_ phase of the T cell cycle and reduction in IFN-γ, IL-2, and TNF-α production (6). MSCs can also suppress many other T cell mediators (14,15,17).

Several clinical studies investigated the hypoimmunogenic and immunosuppressive nature of allogeneic MSCs. Transplantation of allogeneic MSCs showed favorable results in patients with osteogenesis imperfecta (18), metachromatic leukodystrophy (19), and severe idiopathic aplastic anemia (20). They were also used for graft vs host disease prevention in the transplantation of hematopoietic stem cells (21). Therefore, identification of MSCs’ immunomodulatory properties may facilitate their application in hematology and organ transplantation protocols.

We demonstrated that tonsil-derived MSCs suppressed the growth of T-lymphocytes and secretion of two T-lymphocyte-specific cytokines. However, MSCs have also been shown to impact other T helper subsets involved in diseases characterized by immune dysregulation (such as IL-17-producing T helper 17 or FoxP3-expressing regulatory T cells) (22). Additionally, MSCs suppress DC maturation, and inhibit B cell proliferation, differentiation, and chemotaxis. Therefore, additional studies are required to completely understand the hierarchy of tonsil-derived MSC-associated immunomodulators and determine whether tonsil-derived MSCs can trigger the harmful T-cell effector behavior (23).

Our study did not include children with histopathological diagnosis or microanatomical architecture other than hypertrophic tonsils. While the existing data show only limited impact of distinct tonsillar histopathology on morphologic changes (hypertrophic changes in palatine tonsil are driven only by the germinal center expansion) (24), further studies are needed to determine the cell-intrinsic properties of palatine tonsil-derived MSCs removed for different non-malignant reasons.

The limitations of our study include a limited insight into T-MSC mechanisms involved in the immune response suppression and selective focus on Th1 and Th2 functions, whereas other populations with defined pathogenic role in human disease (eg, Th17 and regulatory T cells) remained unexplored (22). Recent murine model demonstrated that tonsil-derived MSCs expressed both the membrane-bound and soluble forms of programmed death-ligand 1 (PD-L1), which distinguishes them from MSCs derived from other organs. It also found that T-MSC-derived PD-L1 effectively suppressed Th17 differentiation in autoimmune diseases such as psoriasis (13).

Despite these limitations, our study offers an initial assessment of immunomodulatory properties of tonsil-derived MSCs in human samples. Further research is warranted to better explain the mechanisms behind the observed immunosuppressive effect and to provide a foundation for translational consideration.
